# Sun1 deficiency leads to cerebellar ataxia in mice

**DOI:** 10.1242/dmm.019240

**Published:** 2015-08-01

**Authors:** Jing-Ya Wang, I.-Shing Yu, Chien-Chi Huang, Chia-Yen Chen, Wan-Ping Wang, Shu-Wha Lin, Kuan-Teh Jeang, Ya-Hui Chi

**Affiliations:** 1 Institute of Biotechnology and Pharmaceutical Research, National Health Research Institutes, Zhunan, Miaoli County 35053, Taiwan; 2 Department of Clinical Laboratory Sciences and Medical Biotechnology, National Taiwan University Hospital, Taipei 10048, Taiwan; 3 Center of Genomic Medicine, National Taiwan University Hospital, College of Medicine, National Taiwan University, Taipei 10048, Taiwan; 4 Institute of Molecular and Genomic Medicine, National Health Research Institutes, Zhunan, Miaoli County 35053, Taiwan; 5 Laboratory of Molecular Microbiology, National Institute of Allergy and Infectious Diseases, National Institutes of Health, Bethesda, MD 20892, USA; 6 Department of Laboratory Medicine, National Taiwan University Hospital, Taipei 10048, Taiwan; 7 Graduate Institute of Basic Medical Science, China Medical University, Taichung 40402, Taiwan

**Keywords:** Sun1, LINC complex, Cerebellar ataxia

## Abstract

Migration and organization of the nucleus are essential for the proliferation and differentiation of cells, including neurons. However, the relationship between the positioning of the nucleus and cellular morphogenesis remains poorly understood. Inherited recessive cerebellar ataxia has been attributed to mutations in *SYNE1*, a component of the linker of nucleoskeleton and cytoskeleton (LINC) complex. Regardless, *Syne1*-mutant mice present with normal cerebellar development. The Sad1-Unc-84 homology (SUN)-domain proteins are located at the inner nuclear membrane and recruit Syne proteins through the KASH domain to the outer nuclear membrane. Here, we report an unrecognized contribution of Sun1 and Sun2 to the postnatal development of murine cerebellum. Mice depleted of *Sun1* showed a marked reduction in the cerebellar volume, and this phenotype is exacerbated with additional loss of a *Sun2* allele. Consistent with these histological changes, *Sun1^−/−^* and *Sun1^−/−^Sun2^+/−^* mice exhibited defective motor coordination. Results of immunohistochemical analyses suggested that Sun1 is highly expressed in Purkinje cells and recruits Syne2 to the periphery of the nucleus. Approximately 33% of Purkinje cells in *Sun1^−/−^* mice and 66% of Purkinje cells in *Sun1^−/−^Sun2^+/−^* mice were absent from the surface of the internal granule layer (IGL), whereas the proliferation and migration of granule neurons were unaffected. Furthermore, the *Sun1^−/−^Sun2^+/−^* Purkinje cells exhibited retarded primary dendrite specification, reduced dendritic complexity and aberrant patterning of synapses. Our findings reveal a cell-type-specific role for Sun1 and Sun2 in nucleokinesis during cerebellar development, and we propose the use of *Sun*-deficient mice as a model for studying cerebellar ataxia that is associated with mutation of human *SYNE* genes or loss of Purkinje cells.

## Abstract (precis)

**Summary:** Mice lacking Sun proteins serve as a working model to study SYNE1-associated cerebellar ataxia; they will also be useful in identifying therapeutic targets for neurodegenerative diseases involving Purkinje cell loss.

## INTRODUCTION

The positioning of the nucleus affects the proliferation, migration and differentiation of neurons (Higginbotham and Gleeson, 2007; Morris et al., 1998; Zhang et al., 2009). Most neurons arise in the germinal zone of the lateral ventricles and migrate toward the pial surface in an inside-out manner. Two processes that affect the movement of neurons are interkinetic nuclear migration and nucleokinesis (Burke and Roux, 2009). Interkinetic nuclear migration involves cell-cycle-dependent movement of the nucleus within the cell from the apical to basal regions (i.e. during G1-S phase), before returning to the apical region in preparation for mitosis (Kosodo et al., 2011). By contrast, nucleokinesis involves movement of the neuron in three steps: neurite extension, migration of the microtubule organization center (MTOC) and finally repositioning of the nucleus (Tsai and Gleeson, 2005). Both interkinetic nuclear migration and nucleokinesis require proper coordination and connection with the cytoskeletal structure and the nucleus for accurate nuclear positioning. Although the majority of neuronal migration in the mammalian brain occurs during the embryonic stage, substantial migration also occurs postnatally. Understanding the mechanisms that initiate, maintain and terminate neuronal migration is key to mapping brain circuitry, and to gaining insight into both normal and pathological neurodevelopment (Ghashghaei et al., 2007).

Mammalian linker of nucleoskeleton and cytoskeleton (LINC) complexes comprise Sad1-Unc-84 homology (SUN)-domain proteins and Klarsicht/ANC-1/Syne homology (KASH)-domain-containing Syne proteins, which mechanically couple the nucleus and cytoskeleton (Chi et al., 2007; Crisp et al., 2006; Haque et al., 2006; Ostlund et al., 2009; Padmakumar et al., 2005). C-termini of Sun proteins are embedded in the inner nuclear membrane (INM) with a trimeric coiled-coil that predisposes them to bind to three KASH peptides in the perinuclear space (Sosa et al., 2012). The N-termini of Sun proteins engage with nuclear lamins, which comprise the nucleoskeleton (Chen et al., 2012, 2014; Chi et al., 2007; Crisp et al., 2006; Haque et al., 2006; Padmakumar et al., 2005). Loss of function in this connection has been shown to affect mammalian development in a tissue-dependent manner (Lei et al., 2009; Yu et al., 2011; Zhang et al., 2010, 2007, 2009), and the nervous system seems to be particularly affected. For example, *Lmnb1*-deficient (*Lmnb1^Δ/Δ^*) and *Lmnb2^−/−^* mice show defective neocortical layering (Coffinier et al., 2011; Vergnes et al., 2004). The proteins Syne1 and Syne2 play crucial roles in anchoring synaptic and non-synaptic myonuclei, which are important for the proper innervation of motor neurons (Zhang et al., 2010, 2007). It appears that Sun1 and Sun2 form redundant complexes with Syne2 to regulate interkinetic and radial neuronal migration in the cerebral cortex (Zhang et al., 2009).

Knockout of the *Sun1* gene in mice causes infertility and impairs telomere attachment to the nuclear envelope, resulting in persistent double-stranded DNA breaks, as well as inefficient homologous pairing and synapse formation in meiosis (Chi et al., 2009; Ding et al., 2007). Interestingly, unlike *Sun1^−/−^* animals, *Sun2^−/−^* mice have no grossly discernible abnormalities and are reproductively normal (Lei et al., 2009), suggesting that the two proteins do not simply serve redundant roles. However, mice that have a homozygous deficiency of both *Sun1* and *Sun2* do not survive birth and show skeletal muscles with destructive myonuclear positioning in the syncytial cells, as well as abnormal lamination in the cerebral cortex (Lei et al., 2009; Zhang et al., 2009).

**TRANSLATIONAL IMPACT**

**Clinical issue**

Nucleokinesis (movement of the nucleus) is a key process that influences neuron migration during embryonic and postnatal development. It requires proper coordination of different proteins that are associated with the microtubule or actin component of the cytoskeleton. The mammalian linker of nucleoskeleton and cytoskeleton (LINC) complex has been shown to mechanically couple the nucleus and the cytoskeleton for migration of the nucleus. It comprises Klarsicht/ANC-1/Syne homology (KASH)-domain-containing Syne proteins (such as Syne1 and Syne2) and the SUN-domain proteins, including Sun1 and Sun2 – which form redundant complexes with Syne2 to regulate neuronal migration in the cerebral cortex. Mutations in SYNE1 have been associated with inherited autosomal recessive cerebellar ataxia type 1 (ARCA1) in humans. However, development of cerebellum is normal in *Syne1^−/−^* mice, which prevents the application of this mouse model to the study of this human disease.

**Results**

In this study, the authors generated genetically modified mice that lack *Sun1* and/or *Sun2* in different allelic combinations, and they report that Sun1 and Sun2 contribute to the development of murine cerebellum. They show that Sun1 is highly expressed in cerebellar Purkinje cells and recruits Syne2 to the nuclear periphery. Sun1 and Sun2 have a dosage-dependent but non-redundant effect on the migration and dendritic morphogenesis of Purkinje cells. Mice deficient for *Sun1* show a marked reduction of the cerebellar volume, and this phenotype is exacerbated with additional loss of a single *Sun2* allele. Consistent with the histological findings on cerebellar alterations, *Sun*-deficient mice exhibit defective motor coordination.

**Implications and future directions**

This study shows that Sun1 and Sun2 play an important role during cerebellar development, in particular in regulating nucleokinesis in Purkinje cells. In view of the functional linkage between Sun and Syne proteins, the *Sun*-deficient mice generated here represent a promising model to study human SYNE-associated cerebellar ataxia. In addition, they can be used to identify potential therapeutic targets for intervention in cerebellar ataxia, as well as in neurodegenerative diseases associated with Purkinje-cell loss.

Mutations in *SYNE1* are associated with human autosomal recessive cerebellar ataxia [e.g. autosomal recessive cerebellar ataxia type 1 (ARCA1) or autosomal recessive spinocerebellar ataxia-8 (SCAR8)]. Intronic *SYNE1* mutations in these individuals affect gene splicing, which can result in the premature termination of the proteins and a loss of the KASH domain. Curiously, *Syne1* knockout mice do not appear to recapitulate the human ARCA1 pathological phenotypes, thereby precluding these mice as a useful animal model (Zhang et al., 2009). We have previously generated *Sun1^−/−^* mice. The Sun1 deficiency affects mammalian gametogenesis and hearing (Chi et al., 2009; Horn et al., 2013). The *Sun1^−/−^* mice in our previous study appeared normal at birth and showed no apparent pathologies, except for infertility (Chi et al., 2009). However, in long-term follow up, ataxic movements were observed in many of these *Sun1^−/−^* mice, which is suggestive of cerebellar dysfunction. Here, we report our findings on the selective defect in the nuclear positioning and primary dendrite specification of *Sun*-deficient Purkinje cells, which resulted in ataxia-producing cerebellar dysmorphogenesis. Thus, *Sun*-deficient mice provide a model for understanding the mechanistic basis of Purkinje-cell-loss-associated ataxias and cerebellar function.

## RESULTS

### *Sun1-*deficient mice demonstrate defective cerebellar development

**Fig. 1.**
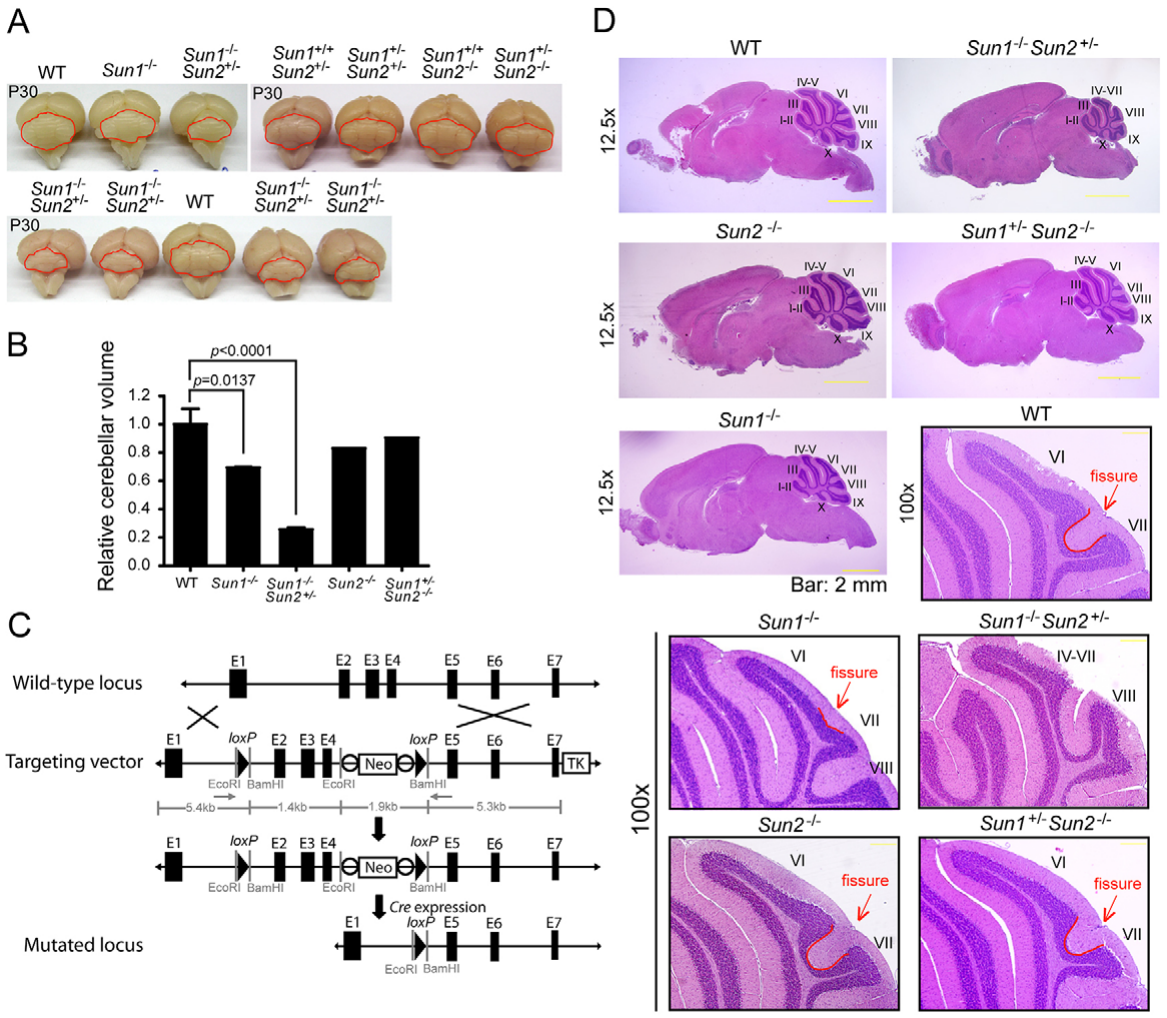
***Sun1^−/−^* mice show aberrant cerebellar development.** (A) Pictures of mouse brains from 30-day-old mice with the indicated genotypes. The mouse cerebellums are outlined in red. (B) Statistics of relative cerebellum volume of the brains shown in A, calculated by using width×length^2^. The volume of cerebellums from *Sun1^−/−^* (*P*=0.0137) and *Sun1^−/−^Sun2^+/−^* (*P*<0.0001) mice is significantly reduced compared to that of WT. Student's *t*-test. (C) Generation of *Sun2*-knockout mice. The WT allele, the targeting vector and the mutated locus of *Sun2* are presented in the scheme. The targeting vector contains the PGK-*Neo* gene (*Neo*) and the thymidine kinase gene (*TK*). The solid triangles represent *loxP* sites, and white circles are *FRT* sites. (D) H&E-stained sagittal sections of paraffin-embedded mouse brains from 30-day-old WT, *Sun1^−/−^*, *Sun1^−/−^Sun2^+/−^*, *Sun2^−/−^* and *Sun1^+/−^Sun2^−/−^* mice. Image magnifications (12.5× and 100×) are indicated. Numerals indicate the lobules, and the fissures between lobule VI and VII of cerebellums are outlined in red lines and indicated by red arrows. Scale bars: 2 mm (200 μm in magnification images).

Through systematically phenotyping organ systems in *Sun1^−/−^* mice, we discovered that the average weight of the brains of adult *Sun1^−/−^* (*n*=12, 402.0±7.2 mg) mice was significantly lower than wild-type (WT) counterparts (WT, *n*=10, 449.2±5.1 mg, *P*<0.001; supplementary material Fig. S1A,B). *Sun1^−/−^* compared with WT mice showed a ∼25% reduction in cerebellar volume (based on measurements of the width and height of the cerebellum; Fig. 1A,B). These findings are remarkable because the average body weights did not differ between *Sun1^−/−^* and WT animals (WT: 24.4±0.653 g, *n*=31 vs. *Sun1^−/−^*: 25.6±1.06 g, *n*=20, *P*=0.3378; supplementary material Fig. S1C).

*Sun1^−/−^Sun2^−/−^* double-null mice show profound growth retardation with severe multi-organ abnormalities and a failure to thrive at birth (Lei et al., 2009; Zhang et al., 2009). In *Sun1^−/−^Sun2^−/−^* mice, lamination of the cerebral neocortex is severely impaired owing to defective neuronal migration (Zhang et al., 2009). To study these observations in greater detail, we generated a new *Sun2*-conditional-knockout mouse (Fig. 1C; supplementary material Fig. S1C-E), in which exons 2 to 4 of *Sun2* were deleted. Consistent with a previous report (Lei et al., 2009), whole-body depletion of *Sun2* (i.e. *Sun2^−/−^*) did not lead to any distinguishing somatic organ abnormalities or changes in body weight, compared with WT mice (*P*=0.3185; supplementary material Fig. S1C). Our *Sun1^−/−^Sun2^−/−^* mice did not survive birth and therefore could not be studied for postnatal development. By contrast, when we bred our mice to create *Sun1^−/−^Sun2^+/−^* animals, this genotype did thrive postnatally, and the average body weight (21.59±0.866 g, *n*=21; supplementary material Fig. S1C) of these mice was only slightly less than that of WT controls (*P*=0.0108 comparing *Sun1^−/−^Sun2^+/−^* with WT). *Sun1^+/−^Sun2^−/−^* mice also appeared to be normal at birth, and the average body weight of these animals (24.25±0.726 g, *n*=16) was not statistically different (*P*=0.8797) from that of WT mice. In observations of gross anatomy, *Sun1^−/−^* and *Sun1^−/−^Sun2^+/−^*, but not *Sun1^+/−^Sun2^−/−^*, mice showed a marked decrease in cerebellar size compared with WT cohorts (Fig. 1A,B). Hematoxylin and eosin (H&E) staining revealed a difference in the foliation of cerebellum in neonatal *Sun1^−/−^* mice (supplementary material Fig. S1F). An absence of the intercrural fissure between lobules VI and VII (Fig. 1D, indicated by red arrows and red outlines) was significant in postnatal day (P)30 *Sun1^−/−^* mice. Moreover, foliation and fissuration of the cerebellar cortex was severely reduced in *Sun1^−/−^Sun2^+/−^* mice compared with WT animals (Fig. 1D). These results suggest discrete, non-redundant roles for *Sun1* and *Sun2* in cerebellar development.

### *Sun1^−/−^* mice suffer from impaired motor coordination

**Fig. 2.**
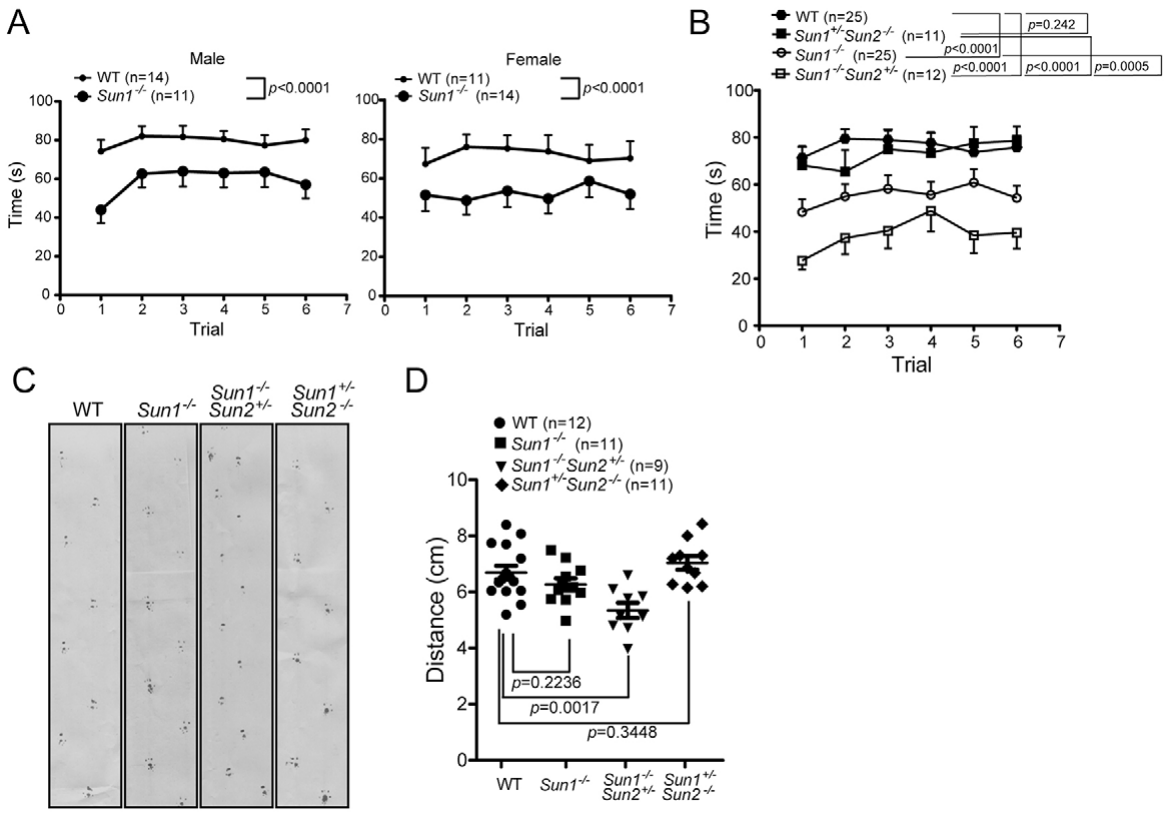
**The coordinated movement of mice deficient for *Sun1* and/or *Sun2*.** (A) Motor coordination of 3- to 4-month-old WT and *Sun1*^−/−^ mice was tested using rotarod. The number of mice tested for each gender are indicated. The time to fall off the rotarod (latency) was recorded. (B) Similar to A, the time to fall off the rotarod from age-matched (3-4 months old) animals with genotypes WT, *Sun1*^−/−^, *Sun1*^+/−^*Sun2^−/−^*, and *Sun1*^−/−^*Sun2^+/−^*. Mean±s.e.m. for each genotype is presented. (C,D) Footprint analysis obtained from 5- to 6-month-old WT, *Sun1*^−/−^, *Sun1*^+/−^*Sun2^−/−^*, and *Sun1*^−/−^*Sun2^+/−^* animals. (D) Footprint analysis statistics and the mean±s.e.m. for each genotype is presented. Each data point represents the averaged stride length from 5 or 6 steps of each mouse. Student's *t*-test.

The histological differences described above and our observations of the behavior of *Sun1^−/−^Sun2^+/−^* mice (supplementary material Movies 1, 2) prompted us to investigate whether the Sun proteins are involved in cerebellar motor function. The rotarod test is an established method for evaluating motor coordination and balance in mice. Six rotarod trials were performed on age-matched (3-4 months old) WT, *Sun1^−/−^*, *Sun1^−/−^Sun2^+/−^* and *Sun1^+/−^Sun2^−/−^* mice, and the duration that the mice remained on the rod was recorded. No differences in gender were observed in the performance profiles (Fig. 2A); however, the motor coordination of *Sun1^−/−^* (*P*<0.0001) and *Sun1^−/−^Sun2^+/−^* (*P<*0.0001), but not *Sun1^+/−^Sun2^−/−^* (*P*=0.242), animals differed significantly from that of WT controls (Fig. 2B). A comparison of *Sun1^−/−^Sun2^+/−^* and *Sun1^−/−^* mice revealed that the former exhibited a worse motor coordination profile (*P*=0.0005) than the latter (Fig. 2B). The motor functions of our mice were further tested using footprint analysis. *Sun1^−/−^Sun2^+/−^* mice exhibited significant motor impairment, as demonstrated by a reduced length of the hindlimb stride compared with that of the WT cohort (*P*=0017; Fig. 2C). These results indicate that motor coordination is correlated with the extent of histological changes in the cerebellum of *Sun1*- or *Sun2*-deficient mice (Fig. 1).

### Differential contributions of Sun1 and Sun2 to the localization of Syne2 to the nuclear envelope in Purkinje cells

**Fig. 3.**
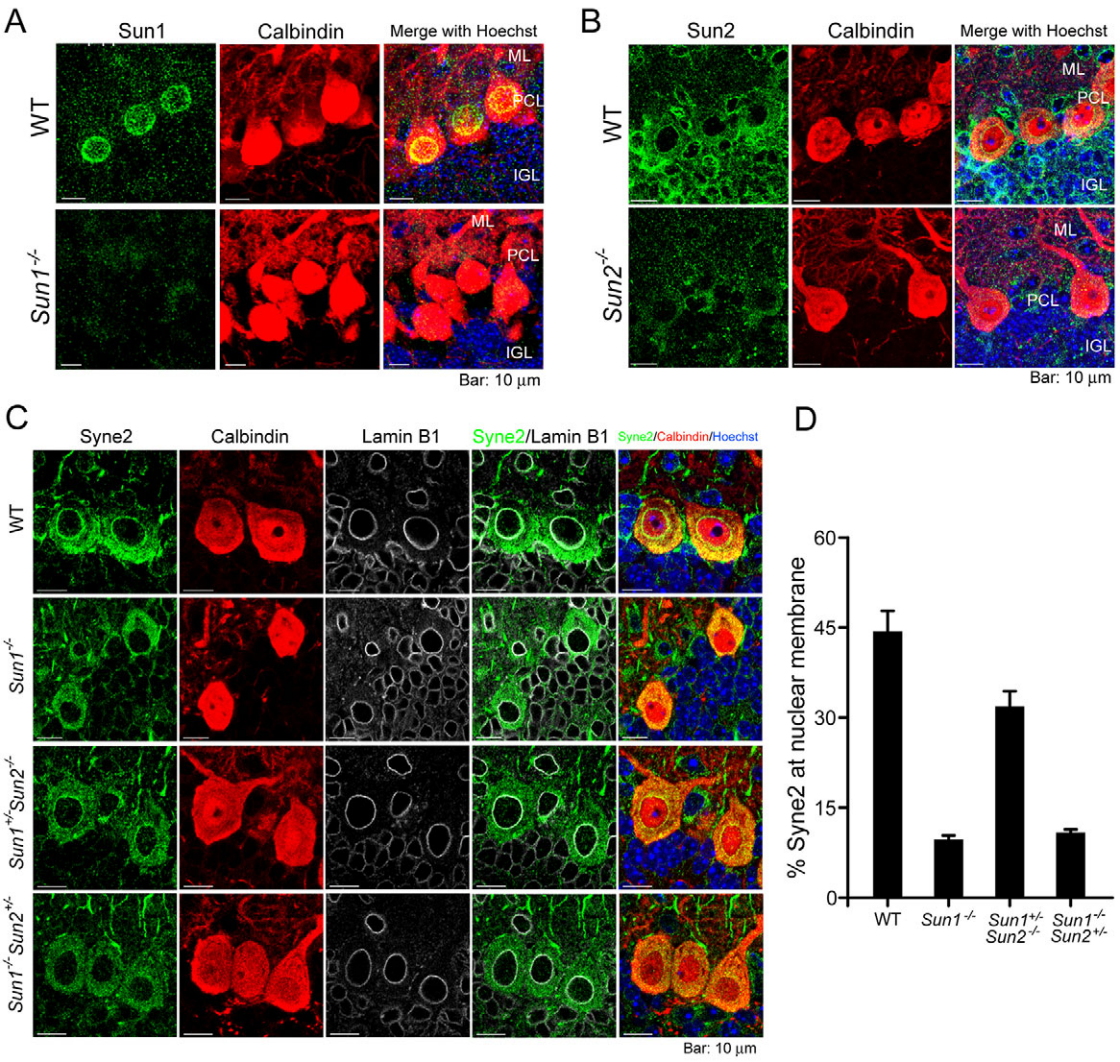
**Sun1 recruits Syne2 to the nuclear membrane.** (A) Expression and localization of Sun1 (green) in 14-day-old mouse cerebellum. The antibody against Sun1 specifically stained the nuclear rim of Purkinje cells. Parallel immunohistochemical staining of Sun1 in *Sun1^−/−^* cerebellum was used as a negative control. (B) Expression and localization of Sun2 (green) in 14-day-old mouse cerebellum by immunohistochemical staining. Parallel immunostaining of Sun2 in *Sun2^−/−^* cerebellum was used as a negative control. DNA was stained by Hoechst33342 (blue). ML, molecular layer; PCL, Purkinje cell layer; IGL, inner granule layer. (C) Expression and localization of Syne2 (green) in 14-day-old mouse cerebellum by immunofluorescent staining using paraffin-embedded tissue sections. Calbindin (red) and lamin B1 (gray) were co-immunostained to mark Purkinje cells and the nuclear membrane, respectively. (D) Percentage of Syne2 at the nuclear membrane. Intensities of fluorescence of Syne2 at the (nuclear membrane)/(total cells) were quantified by ImageJ. Eight to ten cells were quantified for each genotype. Mean±s.e.m. Scale bars: 10 μm.

The cerebellar cortex of mature mice contains three well-defined cell layers surrounding the white matter and deep nuclei – the molecular layer, the Purkinje cell layer and the inner granule layer (IGL) (Sillitoe and Joyner, 2007). Immunofluorescent staining of WT cerebellum revealed that Sun1 was highly expressed in the nuclear envelope of Purkinje cells (marked by calbindin staining), compared with that in interneurons in the molecular layer or internal granular neurons in the IGL (Fig. 3A). By contrast, Sun2 was ubiquitously stained in all three layers of neurons in the cerebellar cortex (Fig. 3B).

Sun1 has been demonstrated to interact with the KASH domain of Syne proteins in the periplasmic space (Crisp et al., 2006; Ostlund et al., 2009; Sosa et al., 2012). The mammalian brain expresses several Syne isoforms (Zhang et al., 2010), including Syne1 and Syne2. To determine whether the Sun proteins serve to localize Syne isoforms in cerebellum, we examined Syne1 and Syne2 localization in cerebellar cells of *Sun*-deficient mice. Consistent with a previously published report (Gros-Louis et al., 2007), Syne1 immunoreactivity in WT Purkinje cells occurred primarily in the cytoplasm (supplementary material Fig. S2), but not in the nuclear membrane. By contrast, clear staining of Syne2 was observed at the nuclear membrane of WT Purkinje cells; however, this staining was significantly reduced when Sun1 was homozygously knocked out (i.e. in *Sun1^−/−^* and *Sun1^−/−^Sun2^+/−^* mice; Fig. 3C). Fig. 3D shows the relative quantities of Syne2 at the nuclear membrane in Purkinje cells from the indicated genotypes. Compared with WT cells, where 44% of Syne2 was located at the nuclear membrane, in *Sun1*-null cells (i.e. *Sun1^−/−^* and *Sun1^−/−^Sun2^+/−^*), approximately 14% of Syne2 was located at the nuclear membrane, whereas the heterozygous expression of a single *Sun1* allele in a *Sun2*-null background (*Sun1^+/−^Sun2^−/−^*) increased the amount of Syne2 at the nuclear membrane to 32% (Fig. 3D). Thus, localization of Syne2 at the nuclear membrane is affected more by the presence of the *Sun1*-null genotype than by the *Sun2*-null genotype. The different physiological outcomes of cerebellar development in *Sun1^−/−^Sun2^+/−^* and *Sun1^+/−^Sun2^−/−^* mice could be attributed to the differential contributions of Sun1 and Sun2 to the recruitment of KASH-domain-containing Syne2 to the nuclear membrane of Purkinje cells.

### Purkinje cells are positioned aberrantly in *Sun1^−/−^* mice

The mouse cerebrum reaches near maturity at birth; however, the cerebellum continues to grow postnatally (Goldowitz and Hamre, 1998; Millen and Gleeson, 2008). During cerebellar development, the granule cell precursors (GCPs) migrate to and proliferate at the surface of the cerebellum to form the external granule layer (EGL). In mice, the GCPs achieve peak proliferation at approximately P8 (Goldowitz and Hamre, 1998). GCPs subsequently exit the cell cycle, differentiate into mature granule cells and migrate radially, passing the developing Purkinje cells, to form the IGL. Therefore, we sought to determine whether the reduced size of *Sun1^−/−^* and *Sun1^−/−^Sun2^+/−^* cerebellums could be explained mechanistically through increased apoptosis or decreased cell proliferation. To investigate these possibilities, we performed staining of Ki67 (supplementary material Fig. S3A) and a terminal deoxynucleotidyl transferase dUTP nick end labeling (TUNEL) assay (supplementary material Fig. S3B) on P7 littermates. No significant differences were observed between WT, *Sun1^−/−^* or *Sun1^−/−^Sun2^+/−^* cerebellums in the TUNEL or the Ki67 assays. Furthermore, the incorporation rate of BrdU at the EGL was not different between WT and *Sun1^−/−^* mice (supplementary material Fig. S3C,D). These results suggest that increased apoptosis or decreased cellular proliferation does not account for the reduction in cerebellar size in *Sun1^−/−^* or *Sun1^−/−^Sun2^+/−^* mice.

**Fig. 4.**
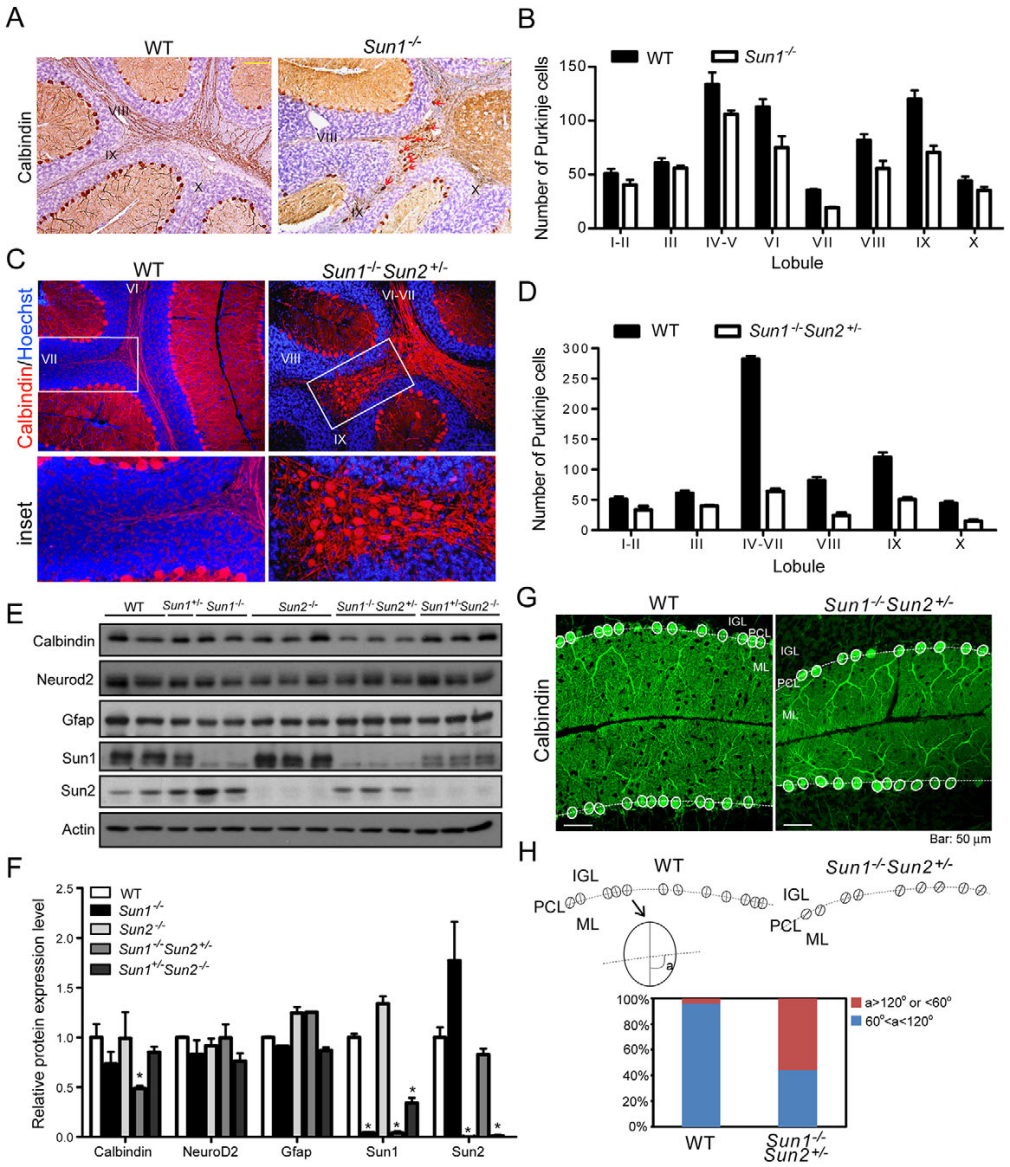
**Aberrant positioning of Purkinje cells in *Sun1*-null cerebellum.** (A) Cerebellum from 30-day-old mouse stained for calbindin (brown) by immunohistochemistry. Some calbindin-positive cells were found in the white matter of *Sun1^−/−^* cerebellum (indicated by red arrows). Numerals indicate the lobules. (B) Quantification of Purkinje cells in each sagittal section at the surface of the IGL in WT and *Sun1^−/−^* cerebellum. Statistics (mean±s.e.m.) were obtained from independent measurements of four mice for each genotype. (C) Calbindin (red) in 30-day-old mouse cerebellum was stained using immunofluorescence. Many calbindin-positive cells were found in the white matter of *Sun1^−/−^Sun2^+/−^* cerebellum (boxed, inset). DNA was stained by Hoechst 33342 (blue). (D) Quantification of Purkinje cells in each sagittal section at the surface of the IGL in WT and *Sun1^−/−^Sun2^+/−^* cerebellum. Statistics (mean±s.e.m.) were obtained from independent measurements of four mice for each genotype. (E) Western blot analysis for protein expression of calbindin, Neurod2, Gfap, Sun1 and Sun2 in cerebellums of adult mice with the indicated genotypes. (F) Quantification of the relative protein expression levels shown in E. Statistics are mean±s.e.m. *P<0.05, Student's *t*-test. (G) Purkinje cells of 30-day-old WT and *Sun1^−/−^Sun2^+/−^* cerebellums were immunostained for calbindin (green). The Purkinje cell bodies are outlined with white ovals. The white dashed lines mark the border between the Purkinje cell layer (PCL) and the inner granule layer (IGL). ML, molecular layer. (H, upper scheme) Schematic presentation of the alignment of the Purkinje cell soma shown in G. The angle (indicated as ‘a’) of the long axis of the soma, and the border (dashed line) between the IGL and ML was measured for each Purkinje cell. (Lower graph) Quantification of ‘a’ in 50 Purkinje cells of each genotype. 96% of WT and 44% of *Sun1^−/−^Sun2^+/−^* Purkinje cells showed ‘a’ between 60° and 120°; and 4% of WT and 56% of *Sun1^−/−^Sun2^+/−^* Purkinje cells showed ‘a’ >120° or <60°. Scale bars: 50 μm.

In the *Sun1^−/−^* and *Sun1^−/−^Sun2^+/−^* cerebellums, the newly generated granule cells in the EGL migrated completely in order to form the IGL, with no residual granule cells remaining at the pial surface (Fig. 1D). Thus, an EGL layer was not observed in the mature *Sun1*^−/−^ or *Sun1^−/−^Sun2^+/−^* cerebellums (Fig. 1D). By contrast, although the newly generated *Sun1^−/−^* Purkinje cells were able to migrate radially from the ventricular zone to localize at the periphery of the cortex underneath the EGL, some of them failed to reach their final destination (supplementary material Fig. S1G). The positions of some *Sun1^−/−^* Purkinje cells were superimposed with the inner granule neurons in P0 cerebellum (supplementary material Fig. S1G, indicated by arrowheads). Furthermore, a large increase was observed in the number of Purkinje cells in the white matter of cerebellums in P30 *Sun1*^−/−^ and *Sun1^−/−^Sun2^+/−^* mice (Fig. 4A-D). Detailed quantification revealed that, compared with WT cerebellums, the number of Purkinje cells at the surface of the IGL was reduced by 33% in *Sun1^−/−^* (Fig. 4B) and 66% in *Sun1^−/−^Sun2^+/−^* mice (Fig. 4D), with lobules IV to VII exhibiting the most significant reductions (Fig. 4B,D). Consistent with the histological findings, protein expression of calbindin, but not Neurod2 (expressed in mature neurons) or Gfap (expressed in radial glia), was significantly reduced in *Sun1^−/−^Sun2^+/−^* cerebellum compared with the WT (Fig. 4E,F). We noted that the expression level of Sun2 in *Sun1^−/−^* cerebellum increased approximately 70% compared with that of the WT (Chen et al., 2012), and increased approximately 90% compared with that of the *Sun1^−/−^Sun2^+/−^* cohorts. These results indicated that Sun2 might partially complement the function of Sun1.

Moreover, for the *Sun1^−/−^Sun2^+/−^* Purkinje cells that were correctly positioned at the surface of the IGL, more than 50% of the somas (Fig. 4G, outlined with the solid white line) appeared to have a distorted orientation with their cell soma oriented (measured as angle ‘a’) non-perpendicularly (defined as >120° or <60°) to the border (marked by white dashed lines) of the Purkinje cell layer and the IGL (Fig. 4H). These results suggest that the aberrant positioning and maturation of Purkinje cells, but not the proliferation or migration of granule cells, is the primary cause of the reduced cerebellar volume in *Sun1-*deficient mice.

### *Sun*-deficient mice have defective dendritic arborization in Purkinje cells

Dendrite arborization specifies neuronal connectivity and integration. Sun proteins interact with Syne proteins to connect the cytoskeletal microtubules and actin, which are important for neuronal plasticity (Cingolani and Goda, 2008; Conde and Cáceres, 2009). Thus, we sought to determine whether, in addition to Purkinje cell loss, defective dendritic morphogenesis occurs in the *Sun1^−/−^Sun2^+/−^* cerebellum.

**Fig. 5.**
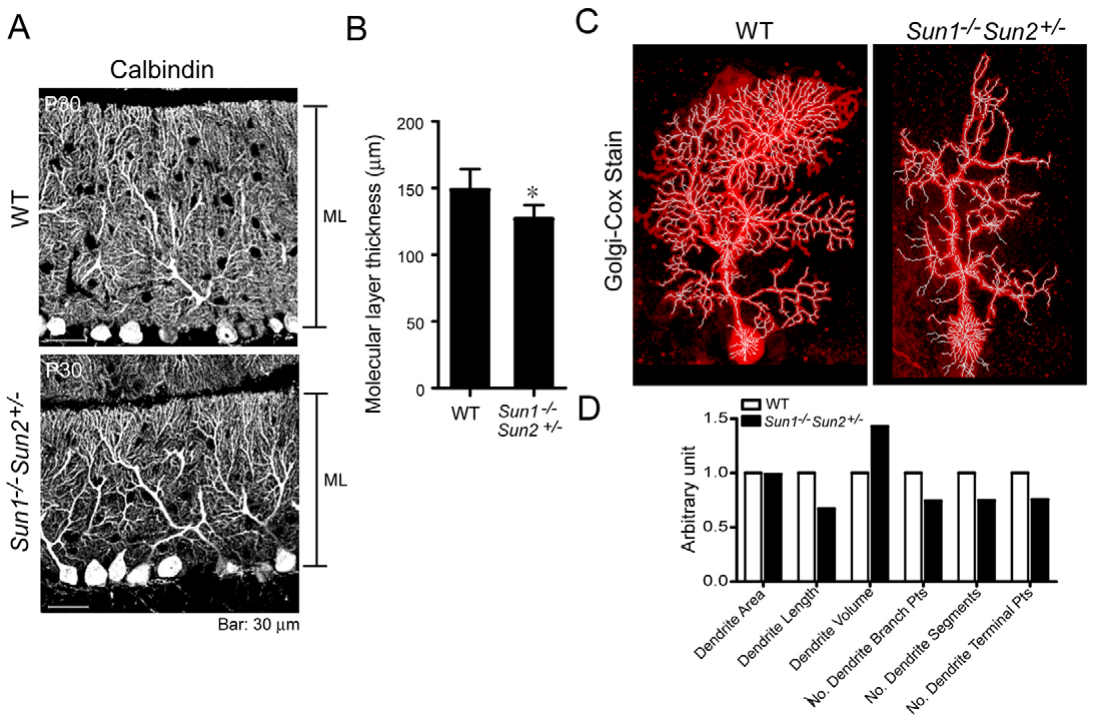
**Mice deficient for *Sun* genes show retarded dendritic arborization.** (A) Immunolabeling of P30 WT and *Sun1^−/−^Sun2^+/−^* Purkinje cell dendrites using an antibody against calbindin (gray). ML, molecular layer. (B) Thickness of the ML in the cerebellum of P30 WT and *Sun1^−/−^Sun2^+/−^* mice shown in A. The mean±s.e.m. was obtained from four mice of each genotype. *P<0.05, Student's *t*-test. (C) Golgi-Cox silver stain (red) of a WT (left panel) and a *Sun1^−/−^Sun2^+/−^* (right panel) Purkinje cell. The total dendritic area, length, volume, segments, branching points, segments and terminal points was analyzed using the Imaris 7.2 Filament Tracer function (white lines). The quantification of the data and statistics are shown in D. Scale bars: 30 μm.

In P30 littermates, in which the cerebellum is mature, we observed a significant reduction in the thickness of the molecular layer in *Sun1^−/−^Sun2^+/−^* cerebellum, compared with that of WT (Fig. 5A,B). Dendritic arborization of a single Purkinje cell was studied using the Golgi–Cox silver-impregnation method (Fig. 5C; Ranjan and Mallick, 2010). Limited by the capricious nature of this technique (Pilati et al., 2008), we compared the dendrite pattern of a WT and a *Sun1^−/−^Sun2^+/−^* Purkinje cell of comparable dendritic area (Fig. 5C,D). The total dendrite length, segments and branching points (analyzed using Imaris 7.2 software) were reduced by approximately 20-30% in the *Sun1^−/−^Sun2^+/−^* Purkinje cell compared with those of the WT cell. These results suggest that Sun1 and Sun2 not only contribute to the positioning of Purkinje cells, but also to the branching of their dendrites.

### Specification of Purkinje primary dendrites and patterning of synapses are affected in *Sun*-deficient cerebellum

**Fig. 6.**
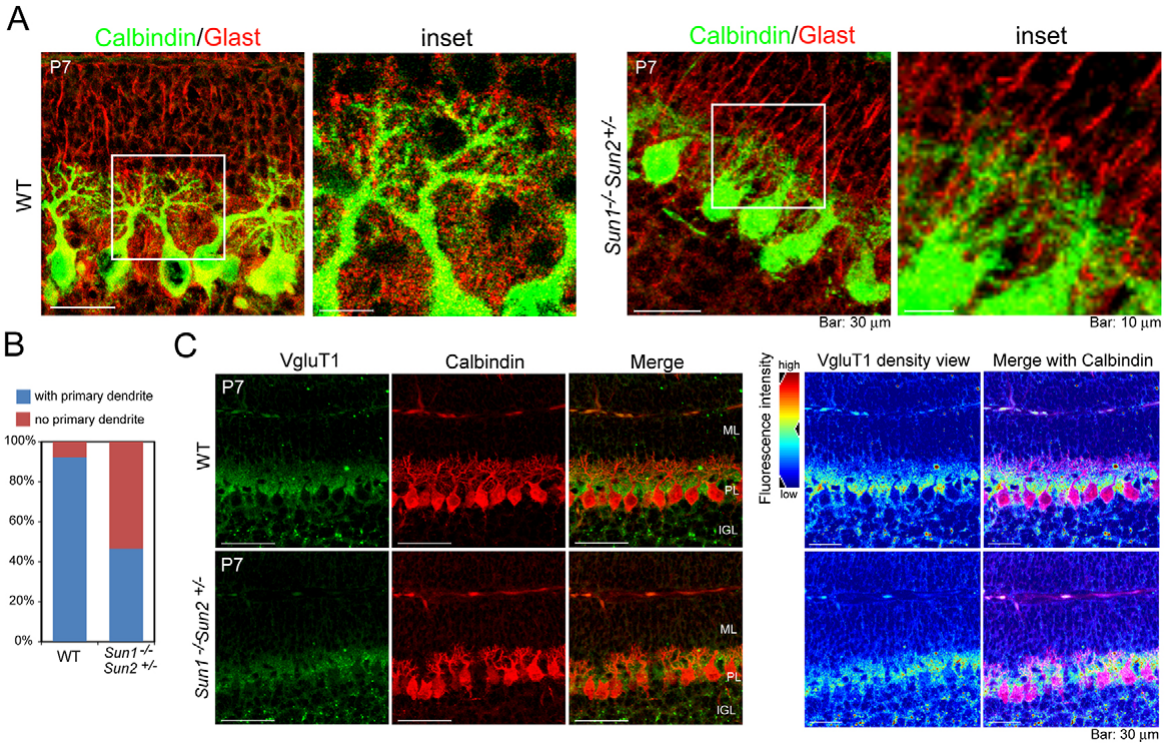
**Aberrant synaptic patterning in the developing cerebellum upon deficiency of *Sun* genes.** (A) Cerebellum sagittal sections from 7-day-old WT and *Sun1^−/−^Sun2^+/−^* mice were immunostained for calbindin (green) to mark Purkinje cells and Glast (red) to label radial glia. (B) Quantification of primary dendrites of P7 WT and *Sun1^−/−^Sun2^+/−^* Purkinje cells, which show promising primary dendritic outgrowth. Approximately 50 Purkinje cells were quantified for each genotype. (C, left panel) Immunofluorescent staining of calbindin (red) and VgluT1 (green) in P7 WT and *Sun1**^−/−^**Sun2^+/−^* cerebellum. (Right panel) Density view of VgluT1. The color code of the expression levels is presented on the left. The ‘warm’ colors represent high expression levels, and the ‘cold’ colors represent low expression levels.

We next determined dendrite morphogenesis in developing cerebellum. In mice, Purkinje cell somas extend multiple dendrites in random orientations during the first postnatal week (supplementary material Fig. S4; Tanaka et al., 2006). A single or a few primary dendrites are determined during the second postnatal week, which extend and branch later, forming the most elaborate dendritic tree among the neurons in the central nervous system (McKay and Turner, 2005). Radial glia provide a structural basis for the directional growth of Purkinje cell dendrites (Lordkipanidze and Dunaevsky, 2005). In WT Purkinje cells, dendrites grew directionally in consonance with radial glia processes [visualized by staining for glutamate astrocyte-specific transporter (Glast), a marker of radial glia; Fig. 6A]. By contrast, the Purkinje cells in *Sun1^−/−^Sun2^+/−^* cerebellum that localized at the IGL surface showed distinctly retarded extension of primary dendrites and dendritic arbor, whereas the radial glia processes formed normally (Fig. 6A). Only 46.6% (27 out of 58) of P7 Purkinje cells in *Sun1^−/−^Sun2^+/−^* cerebellum showed significant extension of primary dendrites compared with 92.3% (48 out of 52) in WT cohorts (Fig. 6B).

In developing murine cerebellum, vesicular glutamate transporter 1 (VgluT1 encoded by the *Slc17a7* gene) that is localized to the excitatory axon terminals of parallel fibers arising from granule cells has been shown to mediate glutamate uptake into synaptic vesicles of Purkinje neurons (Hioki et al., 2003). Vesicular glutamate transporters are crucial for the balance between excitation and inhibition (Fremeau et al., 2004), and are involved in brain disorders, such as schizophrenia, Alzheimer's disease and Parkinson's disease (Harrison et al., 2003; Kashani et al., 2007; Kirvell et al., 2006). Using immunofluorescent staining, we found that VgluT1 was clustered at the interface of the molecular layer and the GCL in P7 WT cerebellum, forming goblet-like structures, surrounding the newly extended primary dendrites of Purkinje cells (Fig. 6C). By contrast, in *Sun1^−/−^Sun2^+/−^* cerebellum, the distribution of VgluT1 was relatively amorphous (Fig. 6C). The aberrant synaptic patterning of VgluT1 suggests that the efficiency of neurotransmission is affected by the retarded morphogenesis of the Purkinje dendritic tree upon deficiency of *Sun1* or *Sun2*.

## DISCUSSION

Early and adult neurogenesis in mammals requires the accurate translocation of neuronal precursors. Genes that are associated with the microtubules or actin cytoskeleton have been shown to non-redundantly influence mammalian brain size through effects on neurite formation, extension and branching, and synaptogenesis (Bond and Woods, 2006; Hoogenraad and Bradke, 2009; Hotulainen and Hoogenraad, 2010). The inner nuclear membrane proteins Sun1 and Sun2 bridge the nucleoskeleton and cytoskeleton, and have been reported to play a role in embryonic neurogenesis (Zhang et al., 2009). In this study, we further demonstrate that Sun1 and Sun2 have a dosage-dependent but nonreciprocal effect on the migration and dendritic morphogenesis of Purkinje cells during postnatal cerebellar development. Unlike *Sun1^−/−^Sun2^−/−^* or *Syne1^−/−^Syne2^−/−^* mice, which suffer from defective neocortical lamination and fail to thrive after birth (Zhang et al., 2009), the viable *Sun1^−/−^* and *Sun^−/−^Sun2^+/−^* animals, which present with cerebellar ataxia, might serve as a working model for the study of the molecular mechanisms underlying *SYNE1*-associated ARCA1 (Dupré et al., 2007; Gros-Louis et al., 2007), as well as for the identification of therapeutic targets in neurodegenerative diseases involving Purkinje cell loss (Millen and Gleeson, 2008). In addition, the *Sun2*-conditional-knockout mouse developed in this study could be further applied in investigations of LINC-associated human diseases and tissue patterning during development.

The nucleus is the most prominent organelle involved in repositioning the soma, also known as nucleokinesis (Tsai and Gleeson, 2005). Neuronal migration is generally initiated by chemotaxis, which triggers a process of elongation and the translocation of the nucleus (Marín et al., 2006). In this study, we showed that Sun1 is selectively expressed in Purkinje cells and mediates the positioning of the nucleus and cellular morphogenesis (Figs 3-5), whereas migration of granule neurons is not affected by the depletion of Sun1 or Sun2 (Fig. 1D). During development, Purkinje cells and granule neurons are generated from two anatomically distinct progenitor zones – the ventricular zone and the more dorsally located rhombic lip, respectively (Millen and Gleeson, 2008; Sillitoe and Joyner, 2007). Upon differentiation, the postmitotic Purkinje cells leave the ventricular zone and migrate radially (inside-out) within the developing anlage. Conversely, cells exiting the rhombic lip migrate over the anlage and proliferate to form the EGL, finally migrating radially through the Purkinje cell layer in an outside-in manner to form the IGL of the mature cerebellum (Millen and Gleeson, 2008; Sillitoe and Joyner, 2007). In *Sun1^−/−^Sun2^+/−^* cerebellum, approximately 66% of the Purkinje cells failed to present at the surface of the IGL, whereas the granule neurons migrated normally (Fig. 1D; Fig. 4C,D). One plausible explanation for the selective interference of Sun1 depletion in the migration of Purkinje cells but not granule neurons is that Sun1 is only required for the inside-out and not outside-in radial migration of neurons. Another possible explanation is that Purkinje cells require stronger pulling forces for the nucleus owing to the large size of their nuclei compared with those of other neurons. Nevertheless, these results indicate that the radial migration of Purkinje cells but not of granule neurons during cerebellar development is dependent on the expression of Sun1.

Isoforms of Syne proteins have been reported to have tissue-specific expression (Rajgor et al., 2012; Randles et al., 2010; Yu et al., 2011; Zhang et al., 2007, 2009). Thus, Sun proteins are responsible for recruiting KASH-domain-containing Syne isoforms but the roles they play might be dependent on the tissue. The failure to detect localization of Syne1 at the nuclear membrane (supplementary material Fig. S2) might be due to variations in the expression of the Syne1 isoforms (Rajgor et al., 2012; Randles et al., 2010). It has previously been reported that singular depletion of either Sun1 or Sun2 does not affect the nuclear membrane localization of Syne1 or Syne2 in the skeletal muscle or retinal cells (Lei et al., 2009; Yu et al., 2011). However, a separate study has demonstrated that Sun1 is required for the recruitment of Nesprin-4 to the outer nuclear membrane in order to maintain the basal localization of the nuclei in outer hair cells (Horn et al., 2013). In this study, we found that Sun1 is selectively expressed in Purkinje cells (Fig. 3A) and is more important than Sun2 in recruiting Syne2 to the nuclear membrane (Fig. 3C,D). The cerebellar phenotypes in *Sun1^−/−^Sun2^+/−^* and *Sun1^+/−^Sun2^−/−^* mice suggest that Sun1 and Sun2 contribute differently to the robustness of the LINC complex in a dosage- and cell-type-dependent manner (Horn et al., 2013; Lei et al., 2009; Yu et al., 2011; Zhang et al., 2009).

In addition to Sun1, Sun2, Syne1 and Syne2, proteins that interact with the LINC complex – such as the nuclear lamins B1 and B2 (encoded by *Lmnb1* and *Lmnb2*, respectively) – are also associated with neuronal migration and brain development. *Lmnb1*-deficient mice (*Lmnb1^Δ/Δ^*) die shortly after birth with immature lungs, abnormalities in bones and layering of neurons in the brain (Coffinier et al., 2011; Vergnes et al., 2004). *Lmnb2^−/−^* embryos develop normally with respect to size, except for the brain (Coffinier et al., 2010). The *Lmnb2^−/−^* cerebral cortex and cerebellum are small, with abnormal layering of neurons. The common features of defective neuronal migration in *Sun1* and *Sun2* double knockout, *Syne1* and *Syne2* double knockout, *Lmnb1^Δ/Δ^*, and *Lmnb2^−/−^* mice indicate that the connection between the nuclear lamina and the cytoskeleton plays a unique role in neurogenesis (Young et al., 2012; Zhang et al., 2009).

Most eukaryotic cells are spatially asymmetric or polarized. Cell polarity is determined by the geometry of the nucleus, the MTOC, intracellular organelles and the cytoskeleton. Cytoskeletal microtubules and actin filaments are active players during initial neuronal polarization (Bryant and Mostov, 2008; Hoogenraad and Bradke, 2009). Specification of primary dendrites of cerebellar Purkinje cells has been shown to be regulated by cell polarity (Tanabe et al., 2010). Therefore, it would be not surprising if compromising the link between the nucleoskeleton and cytoskeleton through the LINC complex results in retarded primary dendrite specification (Fig. 6A,B). The phenotype in *Sun1^−/−^* and *Sun1^−/−^Sun2^+/−^* cerebellum is similar to that of *reeler* mutants, the neurons of which do not recognize their proper localization and orientation upon completing the migration pathway (Falconer, 1951; Niu et al., 2008; Tissir and Goffinet, 2003). In the *reeler* brain, normal neuronal classes are formed, connections between axons and dendritic trees present no serious defects, and gliogenesis and myelination are not directly altered. However, signal transduction that is mediated by cell-cell interaction is disturbed, as well as the expression of cell-cell interaction molecules (Tissir and Goffinet, 2003). The reduced expression of reelin is associated with schizophrenia and bipolar disorder (Impagnatiello et al., 1998). Intriguingly, a large-scale genome-wide association analysis of bipolar disorder identified *SYNE1* as one of the susceptible loci (Psychiatric GWAS Consortium Bipolar Disorder Working Group, 2011). Therefore, future research addressing whether mice that are deficient in *Sun1* and/or *Sun2*, or *Syne1* and/or *Syne2* show cognition phenotypes such as schizophrenia or bipolar disorder could be beneficial in the field of psychiatric medicine.

## MATERIALS AND METHODS

### Generation of *Sun1-* and *Sun2*-knockout mice

The *Sun1^−/−^* animals were created as described previously (Chi et al., 2009). To generate the *Sun2*-knockout mouse, the conditional knockout vector that targets exons 2-4 of mouse *Sun2* was constructed (Fig. 1C) by cloning a 5.4-kb fragment containing exon 1 of the mouse *Sun2* gene into vector pL253 using the recombineering method (Liu et al., 2003). A *loxP* locus was inserted at the 3′-end of exon 1, followed by a 1.4-kb sequence of the *Sun2* gene containing exons 2-4. A *FRT* locus, *Neo* gene, and a second *FRT* and *loxP* loci were cloned at the 3′ end of exon 4, followed by a 5.3-kb sequence of the *Sun2* gene containing exons 5-7. The *HSV-TK* gene was placed at the 3′-end for negative selection. The constructed *Sun2* target vector was introduced into 129/Sv embryonic stem (ES) cells by using electroporation and were then double-selected using G418 and ganciclovir. Expression of the construct in surviving clones was confirmed by Southern blotting using probes amplified from genomic DNA with primers (Probe 5′-1F: 5ʹ-CAGCACCCTCACATGAGTTGGGT-3ʹ; Probe5′-1R: 5ʹ-TGTACAGGACTGTACTTCCGGCCA-3ʹ). Genomic DNAs extracted from ES cells were digested with *Bam*HI. The WT clones showed a band at 10,800 bp, whereas the successfully integrated clones gave a signal at 8500 bp (supplementary material Fig. S1D). The heterozygous ES cells were electroporated with a vector that expressed Cre recombinase. ES cells that lost exons 2-4 of *Sun2* were confirmed by PCR analyses (supplementary material Fig. S1E) using primers [WT forward (CU): 5ʹ-ATAGTCGACGCATGCTATATGGAGCTGTA-3ʹ; knockout forward (GU): 5ʹ-ATAGTCGACTGGGAACTTCCCATCTCCTC-3ʹ; common reverse (JD): 5ʹ-ATAGCGGCCGCTCCCACTCCATGGTGACCTC-3ʹ]. The PCR product of the WT clones is 489 bp and that of the knockout clones is 795 bp, and their sequences were verified by sequencing. The *Sun2^+/−^* ES cells were injected into C57BL/6 blastocysts. Founder animals that were >90% mosaic were crossbred with C57BL/6 mice, and F1 mice were screened for germline transmission by using PCR. Two successful *Sun2^+/−^* germline-transmitted litters were obtained and were then crossed with WT C57BL/6Nar1 mice to reduce the genetic background of 129/Sv. These *Sun2*-knockout mice (deleted for exons 2-4) differed from other *Sun2*-knockout mice (deleted for exons 11-16 of *Sun2*) that were previously generated by Lei et al. (2009). *Sun2^+/−^* animals were crossed with *Sun1^+/−^* animals to generate *Sun1^+/−^Sun2^+/−^* double haplo-deficient mice, and then *Sun1^+/−^Sun2^+/−^* mice were crossed with *Sun1^+/−^Sun2^+/−^* mice to generate double-knockout animals.

### Immunohistochemistry

Mice were anesthetized and perfused with PBS followed by 4% paraformaldehyde in PBS. Mouse brains were dehydrolyzed and embedded in paraffin. For immunohistochemistry, tissue paraffin sections were deparaffinized using xylene and ethanol. After rehydration, antigen retrieval was achieved by placing the slides in 100°C citrate buffer, pH 6.4 for 50 min. Slides were then rinsed with ddH_2_O and PBS successively. Endogenous peroxides were quenched by 0.5% H_2_O_2_ for 10 min. To prevent non-specific binding, slides were blocked with 1% BSA for 30 min and then incubated with primary antibodies diluted in PBS at room temperature for 1.5 h. After three washes with PBS, a biotin-free MM HRP-polymer (Biocare Medical) was added, followed by development of the color using 3,3′-diaminobenzidine (DAB) substrate-chromogen (Biocare Medical). The nucleus was stained with hematoxylin.

### Immunofluorescent staining and confocal microscopy

Cryopreserved or deparaffinized tissue sections were post-fixed in 4% paraformaldehyde for 30 min at room temperature and permeabilized with 0.5% Triton X-100 in PBS for 30 min at room temperature. To block nonspecific binding, tissues were incubated with 1% BSA or 2% goat serum in PBS for 30 min. Primary antibodies were then applied and incubated for 1.5 h at room temperature. Fluorescence-conjugated (Alexa-488, Alexa-568 or Alexa-633) secondary antibodies were used for detection. Cell nuclei were counterstained with Hoechst 33342 (Invitrogen), and the slides were mounted with Prolong Gold antifade reagents (Invitrogen).

### Antibodies

The mouse anti-Sun1 antibody was raised in rabbit and purified with protein-A agarose as described previously (Chi et al., 2009). The rat anti-BrdU and rabbit anti-Syne1, rabbit anti-Sun2, rabbit anti-VgluT1, rabbit anti-Neurod2 and rabbit anti-Gfap antibodies were obtained from Abcam; the mouse anti-calbindin and mouse anti-β-Actin antibodies were from Sigma-Aldrich; the rabbit anti-Ki67 antibody was from Thermo; the guinea pig anti-Glast antibody was from Millipore; the rabbit anti-Syne2 antibody was from Genetex.

### TUNEL assay

DNA strand breaks were detected by using the TUNEL assay, as described by the manufacturer (Chemicon). Terminal deoxynucleotidyl transferase (TdT) catalyzes a template-independent addition of nucleotide triphosphates to the 3′-OH ends of double-stranded or single-stranded DNA. In brief, perfused brain cryo tissue sections were fixed in 1% paraformaldehyde in PBS at room temperature for 10 min. After two washes with PBS, tissues were post-fixed in precooled ethanol:acetic acid 2:1 for 5 min at −20°C. After another two washes of PBS for 5 min, the tissue specimens were incubated with equilibrium buffer at room temperature for 10 min, followed by application of the working strength TdT enzyme and FITC-conjugated nucleotides in a humidified chamber at 37°C for 1.5 h. The reaction was stopped by using working strength stop/wash buffer, and the nuclei were counterstained with Hoechst 33342.

### Rotarod behavior test

Age-matched animals (3-4 months old) were placed on the rod with a constant rotating speed of 20 rpm in order to get familiarized with the rotarod apparatus (Rotamex-5, Columbus Instruments) 1 day before the testing. On the trial day, animals were tested by increasing the rod speed from 0 to 30 rpm in 30 s, the test then continued at 30 rpm for an additional 60 s. The time to fall off the rotarod (latency) was recorded; animals were then returned to the home cage. Six trials were recorded within 1 day, with an interval of 30 min between each trial. The procedure and use of the animals were approved (protocol number: NHRI-IACUC-098086-A and NHRI-IACUC-098043-A) by the Institutional Animal Care and Use Committee (IACUC) of National Health Research Institutes (NHRI, Zhunan, Miaoli County, Taiwan).

### Hindpaw footprint test

The hind paws of mice at 5-6 months old were dipped into black non-toxic paint. Mice were allowed to walk through a plastic tunnel (9×9×40 cm), the floor of which was covered with a sheet of white paper (9×40 cm). Step length for each mouse was analyzed. The step length is defined as the average distance of 5-6 steps. The procedure was approved by IACUC of NHRI (protocol number: NHRI-IACUC-098043-A).

### Golgi-Cox stain

The Golgi-Cox method for staining neurons in the brain was performed according to the protocol described by Ranjan and Mallick (2010). In brief, brains from adult mice (3-4 months old) were removed and washed with distilled water followed by freshly prepared Golgi-Cox solution (5% sodium dichromate, 5% mercuric chloride, 5% potassium dichromate). Cerebellums were sliced sagittally (approximately 5-mm thick) and then covered with Golgi-Cox solution and incubated at 37°C in the dark for 7 days. At the end of incubation, 200-μm thick sections were prepared from each block using a vibratome (VT1200, Leica). Slices were rinsed twice (5 min each) in distilled water to remove traces of impregnating solution and then processed with the following procedures: dehydrated in 50% alcohol for 5 min, soaked in ammonia solution (3:1, ammonia:distilled water) for 5 min, rinsed twice (5 min each) in distilled water, soaked in 5% sodium thiosulfate for 10 min in dark, rinsed twice for 2 min each in distilled water, and dehydrated successively twice (10 min each) in 70%, 80%, 95%, and 99.5% ethanol, cleared in xylene, and mounted in Permount mounting medium (Surgipath) on glass slides. Images were taken on a Nikon Ti-E microscope, and the images were processed using NIS element or Helicon Focus (Heliconsoft) software.

### Western blotting

Total cell lysates from vermis of mouse cerebellums were extracted using RIPA buffer [50 mM HEPES, pH 7.3, 150 mM NaCl, 2 mM EDTA, 20 mM β-gylcerophosphate, 0.1 mM Na_3_VO_4_, 1 mM NaF, 0.5 mM DTT and protease inhibitor cocktail (Roche Applied Science, Indianapolis, IN)] containing 0.5% NP-40 plus rigorous sonication. The lysates were analyzed by using SDS-PAGE, followed by transfer to polyvinylidene fluoride (PVDF, Millipore) membranes and blotting with primary antibodies. Corresponding alkaline-phosphatase-conjugated secondary antibodies (Sigma-Aldrich) were added, and the blots were developed by chemiluminescence following the manufacturer's protocol (PerkinElmer).

## Supplementary Material

Supplementary Material
