# Supplementary material for: Sun1 deficiency leads to cerebellar ataxia in mice
Source: Dis Model Mech. 2015 Aug 1;8(8):957–67. doi: 10.1242/dmm.019240 (PMC4527285; doi:10.1242/dmm.019240)
Supplement: Supplementary Material [file supp_8_8_957__index.html]

Supplementary Material 

# Sun1 deficiency leads to cerebellar ataxia in mice

## DMM019240 Supplementary Material

- Supplementary Material
