# Supplementary material for: Sun1 deficiency leads to cerebellar ataxia in mice
Source: Dis Model Mech. 2015 Aug 1;8(8):957–67. doi: 10.1242/dmm.019240 (PMC4527285; doi:10.1242/dmm.019240)

Supplementary Figures and Legends

Supplementary Figure 1

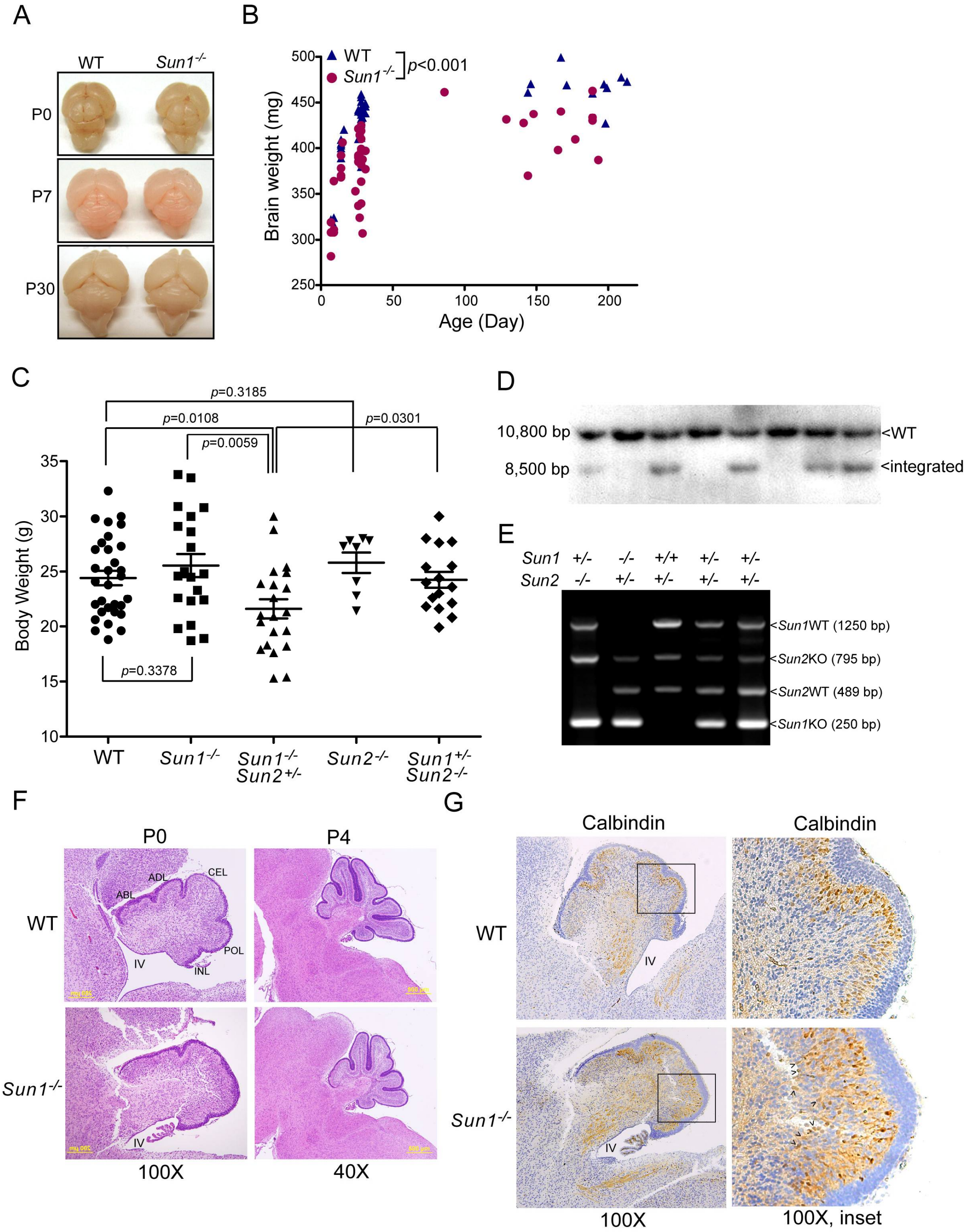

Supplementary Figure S1. Depletion of *Sun1* retards development of mouse cerebellum. (A) Pictures of mouse brains isolated from neoborn (P0), 7 (P7) and 30 (P30) day old WT and *Sun1*$^{-/-}$ mice. Mouse brains were fixed in 4% paraformaldehyde before

the pictures were taken. (B) Brain weights of WT and *Sun1*$^{-/-}$ mice. Each data point represents one mouse at various ages. *P* <0.001 comparing the brain weights of WT and *Sun1*$^{-/-}$ mice that are > 50-day-old, t-test. (C) Body weights of 3-4 month old mice with genotypes WT, *Sun1*$^{-/-}$, *Sun1*$^{-/-}$*Sun2*$^{+/-}$, *Sun2*$^{-/-}$, and *Sun1*$^{+/-}$*Sun2*$^{-/-}$. Each data point represents one mouse. (D) Southern blot of genomic DNAs extracted from *Sun2* knockout ES cells. The WT clones showed a band at 10,800 bp while the successfully integrated clones gave a signal at 8,500 bp. (E) Genotyping of *Sun1* and *Sun2* knockout in mice using DNA PCR. The PCR product of WT *Sun1* is 1250 bp, and the product size of the knockout *Sun1* is 250 bp. The WT *Sun2* allele generated a 489 bp product while the *Sun2* knockout allele generated a 795 bp product. (F) H&E stained sagittal sections of paraffin embedded mouse brains from 0- and 4-day-old WT and *Sun1*$^{-/-}$ mice. Images were taken at 100× and 40× magnifications, respectively. IV, fourth ventricle; ABL, anterobasal lobe; ADL, anterodorsal lobe; CEL, central lobe; POL, posterior lobe; INL, inferior lobe. (G) Immunohistochemical staining of Calbindin for the localization of Purkinje cells in P0 WT and *Sun1*$^{-/-}$ cerebellum. Images were taken at 100× magnification. The arrowheads point to Purkinje cells whose positions are superimposed with inner granule neurons. IV, fourth ventricle.

Supplementary Figure 2

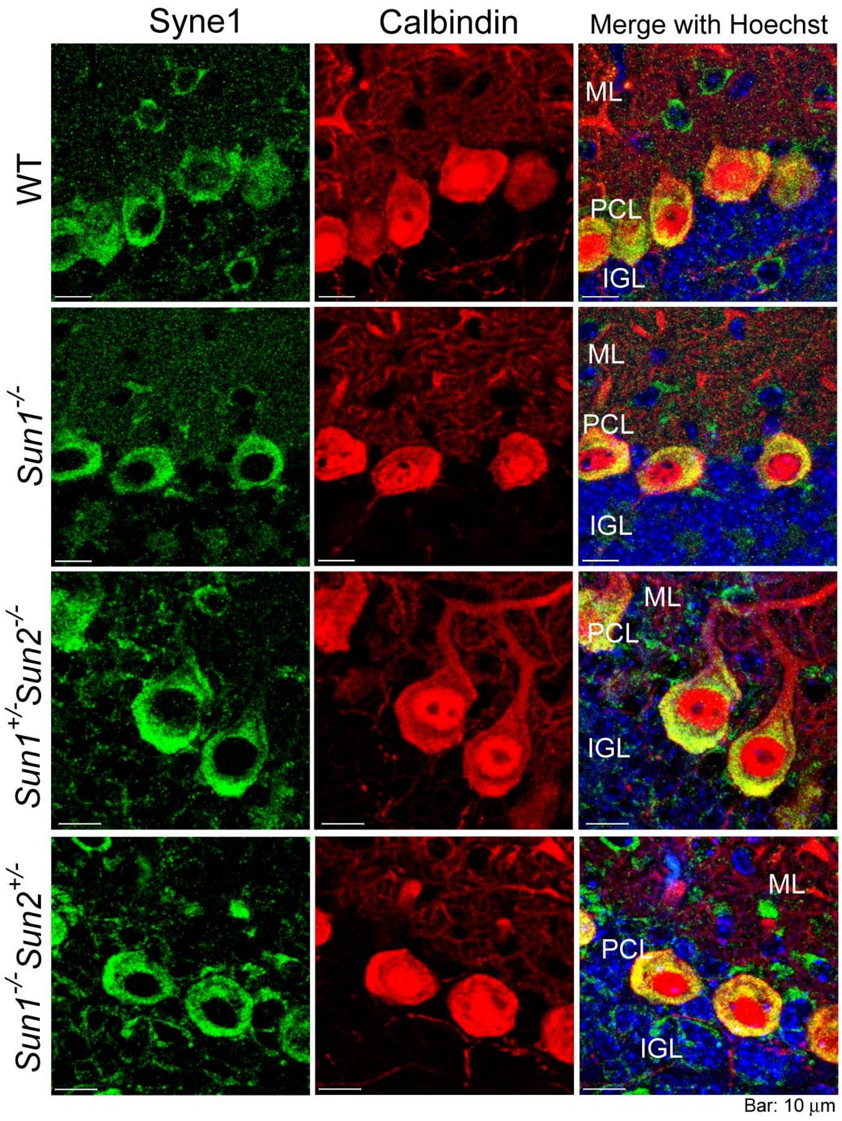

Supplementary Figure S2. Syne1 expression in mouse cerebellum. Cerebellar sagittal-sections from P14 mice with indicated genotypes stained with antibodies to Syne1 (green) and calbindin (red). The yellow signal in the merge picture indicates that Syne1 is expressed in Purkinje cells. However, the Syne1 expression is not concentrated at the nuclear membrane as Syne2 shown in Fig. 3C.

Supplementary Figure S3

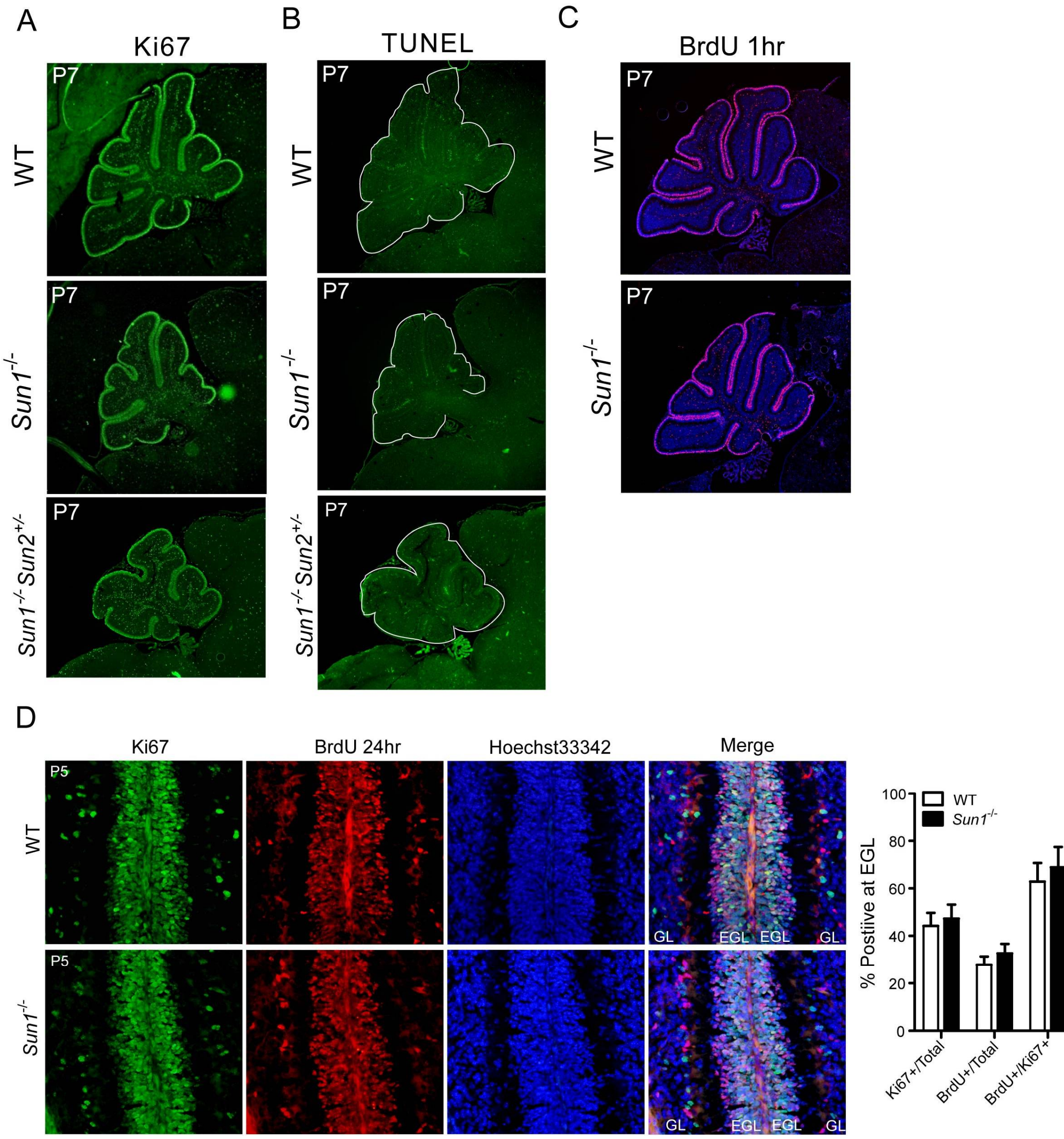

Supplementary Figure S3. Cell proliferation or apoptosis in developing cerebellum was not affected by *Sun1* deficiency. (A) Cell proliferation was assessed by Ki67 (green) immunofluorescence staining in P7 sagittal-sections of WT, $Sun1^{-/-}$ and $Sun1^{-/-}Sun2^{+/-}$ mouse cerebellum. (B) Cellular apoptosis in P7 mice with indicated genotypes was measured by TUNEL assay (green). Sagittal sections of the mouse cerebellums are outlined with white lines. Not many nuclei were positively stained for TUNEL in either WT, $Sun1^{-/-}$ or $Sun1^{-/-}Sun2^{+/-}$ mouse cerebellum. (C) Cell proliferation in P7 mice was assessed by BrdU (50mg/kg) incorporation for 1 hour. Cerebellar sagittal-sections were

stained with an antibody to BrdU (red). No significant difference was observed between WT and *Sun1*$^{-/-}$ cerebellum at EGL. (D) Cell proliferation in P4 mice was assessed by BrdU (50mg/kg) incorporation for 24 hours. Mice were sacrificed at P5. Cerebellar sagittal-sections were stained with antibodies to BrdU (red) and Ki67 (green). Percentages of Ki67-positive nuclei/total nuclei, BrdU-positive nuclei/total nuclei and BrdU positive nuclei/Ki67 positive nuclei at EGL of lobule VI-V were summarized at right. Statistics are mean±SEM from 2 mice.

Supplementary Figure 4

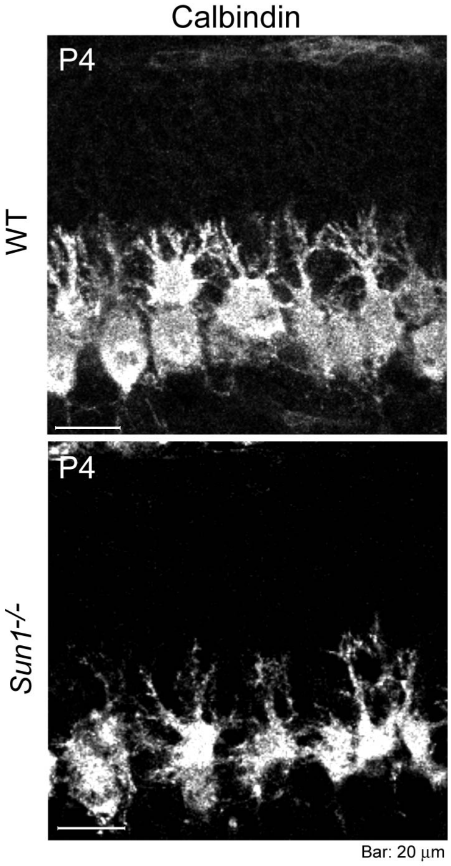

Supplementary Figure S4. Purkinje cell extends multiple dendrites during the first postnatal week. Cerebellar sagittal-sections from P4 WT and *Sun1*$^{-/-}$ mice stained with an antibody to calbindin (grey).

Supplementary Movie Legends

Supplementary Movies. Movement of a WT (movie 1) and a *Sun1*$^{-/-}$*Sun2*$^{+/-}$ (movie 2) mouse at 5-6 months old. The WT littermate appears phenotypically normal while the *Sun1*$^{-/-}$*Sun2*$^{+/-}$ mouse displays ataxia.

Supplementary Movie1

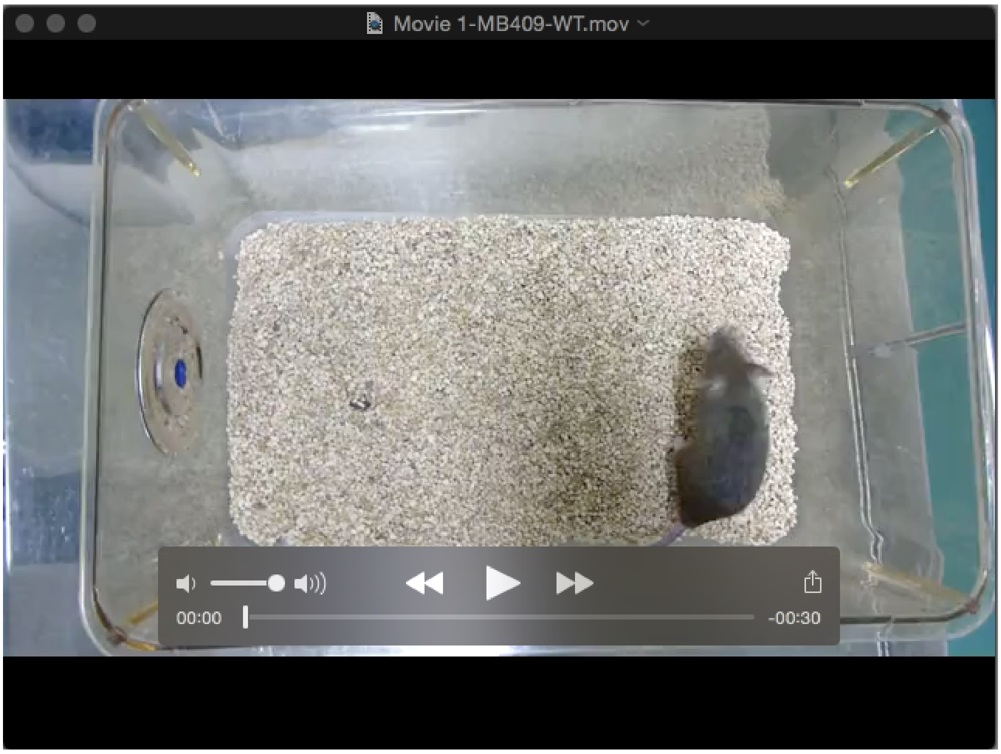

Supplementary Movie2

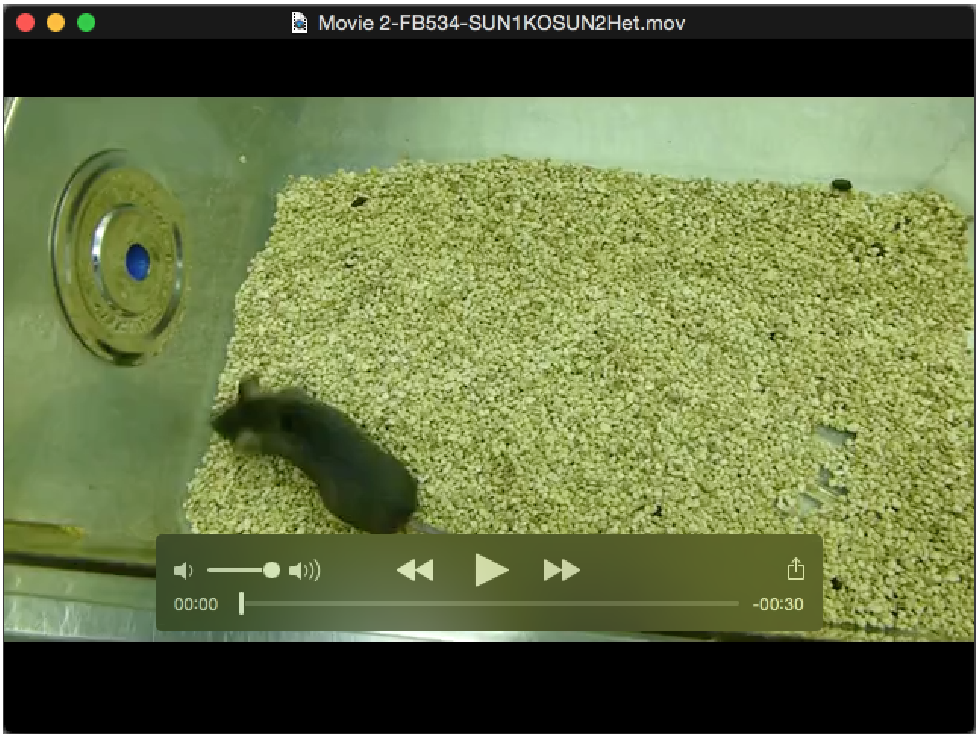

Supplement: Supplementary Material [file supp_019240_DMM019240supp.pdf]
